# Effect of *Ferula persica* plant methanol extract on the level of Cox-2 in induced squamous cell carcinoma (SCC) in rat tongue

**DOI:** 10.15171/joddd.2018.014

**Published:** 2018-06-20

**Authors:** Sepideh Vosoughhosseini, Amirala Aghbali, Parya Emamverdizadeh, Mohammad Razbani, Mehran Mesgari, Ali Barzegar

**Affiliations:** ^1^Professor, Department of Oral and Maxillofacial Pathology, Faculty of Dentistry, Tabriz University of Medical Sciences, Tabriz, Iran; ^2^Associate professor, Department of Oral and Maxillofacial Pathology, Faculty of Dentistry, Tabriz University of Medical Sciences, Tabriz, Iran; ^3^Assistant Professor, Department of Oral and Maxillofacial Pathology, Faculty of Dentistry, Tabriz University of Medical Sciences, Tabriz, Iran; ^4^Postgraduate Student, Department of Oral and Maxillofacial Pathology, Faculty of Dentistry, Tabriz University of Medical Sciences, Tabriz, Iran; ^5^Drug Applied Research Center, Tabriz University of Medical Sciences, Tabriz, Iran; ^6^Assistant Professor, Department of Community Nutrition, Faculty of Nutrition, Tabriz University of Medical Sciences, Tabriz, Iran

**Keywords:** Cox-2, *ferula persica*, squamous cell carcinoma

## Abstract

***Background:*** More than 90% of oral cancers are cases of squamous cell carcinoma. Standard treatment of cancer includes a combination of surgery, chemotherapy and radiotherapy. Each of these treatments, however, brings about certain problems and side effects. Today herbal medicine, has become a more preferable option in dealing with health problems or preventing them because this type of medicine has better compatibility with the body and does not cause undesirable side effects. In this study , the effect of *Ferula persica* plant methanol extraction on Cox-2 levels in SCC induced rat tongue is conducted in vivo.

***Methods:*** In this lab research, 75 rats from SD race in the age – range of 2/5 – 3 months were selected and put in five groups. In order to induce tongue carcinoma, 4– Nitroquinoline 1 (4 NQO) powder was used 3 times a week for each rat. Furthermore, *Ferula persica* extract was given to each of the groups in order to examine Cox-2 changes in the blood.

***Results:*** Comparison of Cox-2 average in various groups resulted in the observation that there was significant difference between the Cox-2 levels in the groups which had only received carcinogen and the other groups. In this group, Cox-2 level was less and in the group that had received Ferula extract (500 mg) along with carcinogen , Cox-2 level was found to be more than other groups.

***Conclusion:** Ferula persica * extract does not have reducing effect on serum Cox-2.

## Introduction

Oral cancer is one of the most common neoplasms in the world. Various factors lead to oral cancers.^1^ More than 90% of oral cancers are cases of squamous cell carcinoma. This type of cancer has the highest mortality rate: almost half of the people suffering from it live more than 5 years after the onset of the disease.^2,3^ In various studies on the etiology of oral cancers, it has been observed that Cox-2 has high expression in oral cancers and precancerous lesions and it seems to be one of the agents involved in the etiology of oral lesions. Cox-2 is an enzyme produced by epithelial cells through stimulation by growth factors, cytokines and mitogens, and results in the production of prostaglandins in response to inflammation, proliferation, cellular differentiation, angiogenesis and metastasis. Increase in Cox-2 expression has been reported in cases of various tumors like colon, lung, bladder and hypopharynx. In recent studies, it has been found out that Cox-2 expression in precancerous lesions and oral cancers is significantly positive. Evidence indicates that use of Cox-2 inhibitors might be a promising method in the treatment of oral cancers.^4-10^

Standard treatment of cancers includes a combination of surgery, chemotherapy and radiotherapy. Each of these treatments has its own side effects and problems. Radiotherapy side effects include dry mouth, sensitivity of the gingivae and teeth, extensive dental caries and problems during swallowing. Chemotherapy side effects are irritation of the mouth or throat, weight loss, thrombocytopenia, infection, nausea, vomiting, loss of appetite and diarrhea. Patients who are in advanced stages of the condition and undergo surgery might suffer from speech disorders, chewing or swallowing problems or facial deformity.^3^

Today in most countries of the world, traditional medicine, especially herbal medicine, is used to prevent or cure diseases. It seems that people are tired of insufficiencies of modern medicine; so they increasingly turn to herbal medicine.^11^

Due to their non-artificial nature and existence of medicinal homologous compounds, traditional medicines have better compatibility with the body; moreover, they do not usually have undesirable side effects. Therefore, these medicines can be useful, particularly in cases when drug use will be prolonged and in chronic diseases.^12,13^ Furthermore, these natural compounds contain antioxidants which are capable of combating cancerous cells. ^11^

Antioxidants protect the body against damage, especially free radicals, and prevent the growth of cancerous cells.^3^ One of the herbs used in conventional medicine is *Ferula persica*. It belongs to *Umbelliferae* family and has 150 varieties all over Asia and Iran.^14^

In traditional medicine, the resin and gum of this plant is used as an expectorant, anti-swelling, anti-bloat, and laxative. Also, it is a cure for neurological disorders, epilepsy and various pains, especially arthralgias.^15,16^ In addition, studies have shown that most *Ferula* varieties have anti-cancerous and antioxidant activity through production of coumarin and umbelliprenin.^17-24^ In some in vitro studies, the anti-cancer effect of *Ferula* extract on cancer cell lines (leukemia, fibrosarcoma, melanoma and breast cancer) has been examined. Alkatib et al^24^ showed that elaeochytrin, a substance present in *Ferula*, is the most effective on human CML cell line (imatinib-resistant) and mice leukemia cell line (dasatinib-resistant), which were effective in densities of 12.4 and 7.8 μM, respectively.^24^

To date, the effect of this herbal extract has not been examined on oral cancers. In previous in vitro studies on the effect of this extract on other cancers, in vivo studies have been suggested for further evaluations.^22^ Since Cox-2 has high expression in cancer and in precancerous lesions of the oral cavity and is an effective agent in carcinogenesis and its inhibitors are considered to be a promising method for cancer` treatment, in this study the effect of methanolic extract of *Ferula persica* on Cox-2 levels in SCC induced in rat tongue was evaluated in vivo for the first time. The results of this study can be used in future research on the etiology, prevention and better treatment – with little side effects – for SCC, the most common type of oral cancer.

## Materials and Methods

### Sample selection and drug prescription method

In this experimental study, based on previous studies, 75 rats (5 groups of 15 rats) of SD race with an age range of 2.5‒3 months and an approximate weight of 200±50 gr were used at a temperature of 22±2°C, 12-hour light cycles and 60±5% humidity.^25-29^ Rats without these prerequisites were excluded from the study. Based on available literature, in order to induce tongue carcinoma, 4-nitroquinolin1-oxide (4NQO) powder (Sigma Co., Germany) was used.^10^

In this study, *Ferula persica* extract was injected to the rats groups A, B, and C. The extract was dissolved in distilled water with different densities of 50, 250 and 500 mg/kg. The carcinogen 4-nitroquinolin -oxide (4NQO) was orally given to rats at the same time. The 4th group, group D, only received the carcinogen and the 5th group, group E, only received the extract through injection in order to study the possible side effects of *Ferula persica*.

### Preparation of Ferula persica water extract

**Figure 1:**
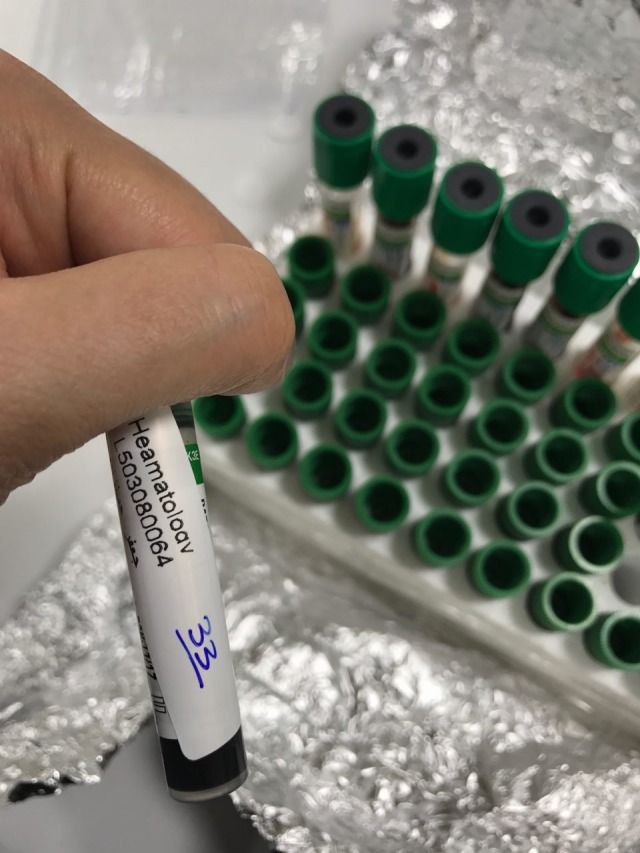
Blood samples of the mice.

This plant was obtained from Khalkhal highlands in Ardebil Province, Iran, and after being checked and verified by the expert in Drug Applied Research Center of Tabriz University of Medical Sciences, it was extracted through Soxhlet extraction method. Since in most of the previous studies, hydroalchoholic extracts of *Ferula* variety plants have been used, the same extract was prepared in this study. To this end, stems, leaves and flowers of this plant were dried at room temperature and then ground to obtain the dry powder. Then its methanolichydroalchoholic extract was prepared through Soxhlet method. The resultant solution was condensed by rotary vacuum evaporator and finally dried on a water bath at a mild temperature of 40°C. The desired density was then prepared from the extract.^25,26^ This was done by solving 500 mg of dry matter in 50 mL of distilled water. Of this solution, 5 milliliter per each kg weight of animal was injected into the animal through intraperitoneal injection (50mg/kg dose); 250 and 500 mg/kg doses were also prepared through dilution with distilled water while paying attention to the results of previous studies that at doses higher than 500 mg/kg motor deficits were observed in rotarod test.^25,30^ At the end of the three-month research period, blood test of the mice was carried out; blood serum was separated and the concentration of Cox-2 of the groups was measured by ELISA method(Figure 1).

Finally, the results were analyzed through Kruskal-Wallis test and Mann-Whitney U test in order to compare Cox-2 levels between different groups.

### Ethical considerations

All the ethical and the humanity considerations were considered and performed according to the Helsinki Declaration during the experiments and euthanasia of the animals. All the animal experiments were approved by the Ethics Committee of the Tabriz University of Medical Sciences.

## Results

In this study, 75 rats (5 groups of 15 rats) of SD race were used; 50,250 and 500 mg/kg concentrations of *Ferula persica* extract dissolved in distilled water were injected through an intraperitoneal technique to the mice groups A, B and C. Carcinogen 4NQO was given orally to the mice at the same time. Group D mice only received carcinogen and group E, received only *Ferula persica* extract in order to study the possible side effects. At the end of the study, biopsies were taken from the rat tongues and the degree of dysplasia in each group was determined. Statistical analysis showed significant differences between groups (P<0.001). Use of *Ferula persica* extract resulted in recovery in groups A, B and C (Table 1).

**Table 1:** Degree of dysplasia in groups A, B, C and D

**Group**	**Type of lesion**					**Total** **No. (%)**
	**Mild Dysplasia** **No. (%)**	**Moderate Dysplasia** **No. (%)**	**Severe Dysplasia** **No. (%)**	**Carcinoma in situ** **No. (%)**	**OSCC** **No. (%)**	
**A**	2 (16.6)	4 (33.3)	3 (25)	1 (8.5)	2 (16.6)	12 (100)
**B**	3 (25)	2 (16.6)	4 (33.3)	2 (16.6)	1 (8.5)	12 (100)
**C**	2 (18.1)	4 (36.3)	3 (27.2)	1 (9.2)	1 (9.2)	11 (100)
**D**	0 (0.0)	0 (0.0)	0 (0.0)	2 (28.5)	5 (71.5)	7 (100)
**Total**	7 (16.6)	10 (23.8)	10 (23.8)	6 (14.2)	9 (21.6)	42 (100)

The outcomes of animals that survived up to the end of the study were separately presented for groups. Average Cox-2 level in each group is presented in the following Table. Cox-2 mean in group C was higher than that in other groups while in group D, it was lower compared to the other groups.

In order to compare Cox-2 means between various groups, Kruskal-Wallis test was used. This test showed significant differences in Cox-2 means between various groups (P=0.038). Comparison of Cox-2 in different groups showed significant differences in Cox-2 levels between group D and other groups (in group D, it was lower and in group C, it was higher than that in other groups) (Table 2).

**Table 2:** Cox-2 levels in groups A , B , C, D and E

**Group**	**Number**	**Mean of Cox-2**	**Std. Deviation**	**Minimum**	**Maximum**
**A**	12	303.083	28.1141	247.5	363.5
**B**	12	297.917	27.4589	250.0	337.0
**C**	11	313.364	26.8133	277.0	351.0
**D**	7	247.214	26.2645	215.0	284.0
**E**	9	305.500	17.4428	274.0	329.5
**Total**	51	296.843	32.2954	215.0	363.5

## Discussion

Today, in many countries traditional medicine, especially herbal medicine, is used to either prevent or cure diseases. These compounds contain antioxidants, which are capable of fighting cancer cells. One of the plants used for this purpose is *Ferula persica* whose antineoplastic effects have been shown in various studies.

In our study, statistical analyses indicated significant differences among the study groups(P<0.001). Following the use of *Ferula persica* extract, recovery was seen in groups A, B and C, a fact which is consistent with other studies in this field. Research has shown that due to production of coumarin and umbelliprenin, most *Ferula* varieties have anti-cancer effects and antioxidant activity.^23,24^ Through a series of in vitro studies, anti-cancer effects of *Ferula* extract on cancer cell lines (leukemia, fibrosarcoma, melanoma and breast cancer) has been studied. Alkatib et al reported that elaeochytrin which a compound in *Ferula* plants has the most cytotoxic effect on human CML cell line and mouse leukemia cell line.^24^

In various studies on oral cancer etiology, it has been observed that Cox-2 is highly expressed in oral cancer and precancerous lesions and it seems that Cox-2 is an agent that plays a role in the etiology of oral lesions.^7-11^

In a study by Arbabi et al on rats suffering from tongue carcinoma, Cox-2 levels increased in blood of rats receiving the carcinogen; topical application of CeleCoxib, Cox-2 selective inhibitor, decreased its level in blood and was therefore considered an adjunct treatment for malignant lesions.^31^

In another study on patients suffering from hypopharyngeal SCC, Ping Pen et al concluded that Cox-2 serum levels increased in these patients and suggested that use of certain Cox-2 inhibitors might be effective in such patients.^8^

Considering these findings in relation to anti-cancer activity of *Ferula* and an increase in Cox-2levels in oral cancer, our study focused on the effect of *Ferula* on Cox-2 enzyme.

Statistical tests showed significant differences in Cox-2 means of various groups (P=0.038). In group D, it was the lowest and in group C, it was the highest. Furthermore, this plant did not decrease Cox-2 levels.

In numerous studies, *Ferula* mechanism in cancer inhibition was examined. For example, in one study, farnesiferol A and galbanic acid extracted from *Ferula* plant inhibited p-glycoprotein transporter in breast cancer cell line resistant to doxorubicin.

Based on this result, they suggested that this plant be considered in studies on chemotherapy drugs for patients suffering from breast cancer resistant to treatment.^32^

In another study, unbelliprenin of Ferula plant had inhibitory effect on cell growth in M4Beu (pigmented malignant metastatic melanoma) through arresting cell cycle in G1 phase and inducing caspase-dependent apoptosis.^33^

In a study by Kim et al,^34^ galbanic acid extracted from *Ferula* plant resulted in the inhibition of angiogenesis as well as proliferation of tumor cells in lung cancer cell lines.

In addition, umbelliprenin and persicalsulphid extracted from *Ferula persica* with low dose have inhibitory effect on tumor cell invasion in fibrosarcoma cell line through inhibition of MMP.

It is suggested that this material be used as an anti-matrixmetalloproteinase in chemotherapy drugs.^23^

In this study, for the first time, the effect of *Ferula persica* plant was examined on Cox-2 level in squamous cell carcinoma (SCC) induced in rat tongue. Biopsy samples indicated that recovery was observed in groups receiving *Ferula persica* extract; statistical analyses showed that *Ferula persica* had no effect on decreasing Cox-2 levels.

## Conclusion

According to the results of this animal research, *Ferula persica* extract did not decrease Cox-2 levels;therefore, it cannot act as a specific Cox-2 inhibitor. In any case, further studies are recommended in this field so that stronger results are obtained and possible anti-cancer mechanisms of *Ferula persica* become known.

## Acknowledgments

This paper was extracted from the thesis No.189, and financially supported by the research council of the Tabriz University of Medical Sciences‏.

## Authors’ contributions

SV, AA, PE, and MR contributed to the concept and the design of the study. MM contributed to the preparation of the extract. AB and MR contributed to the rat experiments. MR drafted the manuscript. All authors contributed to the critical revision of the manuscript, and have read and approved the final paper.

## Funding

This study was funded by Tabriz University of Medical Sciences

## Competing interests

The author declare no competing interests with regards to the authorship and/or publication of this article.

## Ethics approval

This research was approved by the Ethics Committee of the Tabriz University of Medical Sciences.
